# Taggar’s Corundum, Point and Disk Maker

**Published:** 1888-09

**Authors:** 


### EDITORIAL.

### Taggart's Corundum, Point and Disk Maker.

In the early history of dentistry the *tools* used by its votaries were of the crudest character and of the coarsest material, for in those days the manufacture of steel had not reached its present high state of perfection. In order to procure such as would prove available for operations in the mouth and upon the teeth, they were often times forced to make use of tools prepared for other purposes such as shoemakers awls, and others of a like delicate nature. These would be dressed to a proper size and points put upon them as were required, with such instruments the pioneer dentist struggled to serve suffering humanity and right well did they succeed, as an occasional specimen coming under observation even at the present time demonstrates. For drilling out the canals of tortuous roots, they probably used gimlets with which the operation could be accomplished about as successfully as it is done to-day. In this respect we are not much ahead of them. As the higher capabilities of the calling through increasing knowledge became apparent, the inventive genius of its members came to the surface and persevered till at the present day there is not a profession on the face of the globe with a better or more thoroughly equipped armament than the dental. Among the multitude of instruments and appliances, however there are comparatively few, that retain indispensable places of usefulness in our office. Just what the percentage is would be interesting to know, notwithstanding the fact that so many suffer disappointment, from the failure of their products, invention still persists and many useful, aids to our labors are brought forth. Here we wish to refer to an instrument invented and introduced into the profession by Dr. W. H. Taggart, of Freeport, Illinois. Its purpose is to enable the dentist to mould his own Corundum points and disks for the dental engine. Its construction is exceedingly simple, and its operation speedy and gratifying. In fact the molding of a disk is accomplished almost as quickly as the placing of a bur in the engine. The instrument consists of a strong pair of forceps, not much larger than the ordinary extracting forceps—with heavy, flat and broad beaks, between these is placed a circular steel disk with a polished surface so arranged in a socket of one of the beaks as to be self-adjustable to the cutting mold in the other, which is held in its place by a setting screw, and can be removed so as to allow for other forms as may be desired; up through the mold is a small circular hole for the mandrel, which is made of Stubbes steel rod No. 42. To produce the disk, a piece of the ordinary laboratory Corundrum wheel of suitable grit is cut into proper sizes and placed in the mold, then held in the flame of a lamp—preferably a Bunsen—till the shellac becomes quite soft when by gentle compression the blades are brought together and the work is done. It is necessary, of course, to cool the instrument in cold water before attempting to remove the disk. In addition to the ease and simplicity with which this instrument can be used, its economy should recommend it, as a small Corundum costing six cents will produce a set of disks costing in the depots not less than forty to fifty.

W.

---

ONE of the most important processes in operating upon the roots of teeth is that of thoroughly removing the moisture from the canals, for if it is permitted to remain, its tendency is to promote decomposition and thus endanger final success; with the hot air syringes now in use this is almost if not quite impossible for the current of air is so large that it strikes the floor of the cavity and is reflected outwards before it can enter the canal. If the nozzle of the syringe was sufficiently small to pass up the canal to the apex of the root, the desired result might be accomplished, but this would be impracticable, as a moments thought may discern. The only device in use so far as we know that will effectually dry root canals is one invented by Dr. Wooley, of Chicago, and may be described as follows; a long steel handle slightly conical at one end and threaded with a triple slot, for the reception of a broach. This is screwed into a large eliptical copper bulb with a threaded hole through its long axis. The broaches which are made of various degrees of fineness are also made of copper and are extremely pliable and can be passed into the minutest canal without breaking. To use the broach it is placed in the jaws made by the slotting of the handle and screwed into the bulb which fastens the broach securely, the bulb is then held in the flame of a lamp till the required heat is obtained, then passed into the canal, after the surplus moisture has been removed by other means, and held in position till dry. If there is any doubt about the heat being conveyed to the very end of the broach place it, after having been heated, upon the upper eyelid; this will be found to be a very convincing experiment.

